# Quality review of typical value ranges in urodynamic measurements using statistical process control: A single-center retrospective study

**DOI:** 10.14440/bladder.2024.0073

**Published:** 2025-03-27

**Authors:** Xiao Zeng, Hong Shen, Tao Jin, Deyi Luo

**Affiliations:** Department of Urology, Institute of Urology, West China Hospital, Sichuan University, Chengdu, Sichuan 610041, China

**Keywords:** Statistical process control, Urodynamic study, Typical value ranges, Quality review, Continuous variable data

## Abstract

**Background:**

Urodynamic study (UDS) is essential for assessing lower urinary tract function, but quality control methods remain limited. Statistical process control (SPC), a tool originally developed in manufacturing, has shown promise in healthcare for improving quality and reducing variability.

**Objective:**

This study explored the application of SPC to analyze the typical value ranges (TVR) of urodynamic measurements.

**Methods:**

A total of 84 urodynamic traces that met all inclusion criteria were included for analysis. We recorded the TVR for initial intravesical pressure (P_ves_), initial abdominal pressure (P_abd_), and initial detrusor pressure (P_det_) from each enrolled UDS trace. These data were then compared with the standard TVR. In addition, we used the X-bar and S control charts of SPC for process performance analysis.

**Results:**

The study included 20 females and 64 males, with an average age of 58.02 ± 16.09 years. Of the participants, 32 were diagnosed with neurogenic bladder dysfunction, and 52 were diagnosed with non-neurogenic bladder dysfunction. The average TVR for initial P_ves_ was 34.81 ± 10.78 cmH_2_O, P_abd_ 30.92 ± 11.14 cmH_2_O, and P_det_ 4.20 ± 3.73 cmH_2_O. We further analyzed the data using scatter plots. In the X-bar control chart, the control limit (CL) was 22.48, the upper CL (UCL) was 32.04, and the lower CL (LCL) was 12.92. In the S control chart, the CL was 15.78, the UCL was 22.57, and the LCL was 8.9. Two cases exceeded the UCL in the X-bar control chart, and one case exceeded the UCL in the S control chart.

**Conclusion:**

The clinical value of SPC in the quality review of UDS has been confirmed in previous studies. In this study, we preliminarily verified the use of SPC for continuous variable data, such as the TVR of UDS parameters. The results of this study need to be further validated in a larger sample size, multi-center, and prospective study.

## 1. Introduction

Urodynamic study (UDS) is an important method for evaluating the function of the lower urinary tract and is widely used in clinical practice.^1^ As a functional examination technique distinct from imaging techniques, UDS plays a crucial role in the fields of urogynecology and neurourology.^2^ In addition, given the bladder may not always provide reliable information, patients’ complaints may not accurately reflect the true pathophysiological changes in their lower urinary tract.^3^ UDS translate patients’ subjective complaints into objective parameters. Therefore, only well-controlled and high-quality UDS results can assist urologists in making reliable diagnoses.^4^

The International Continence Society initiated a working group to update the Good Urodynamic Practice (GUP) guidelines.^1^ This initiative aimed to gather new evidence and information on urodynamic practice and quality control.^5^ However, the quality review approach for UDS remains limited. Liao and Schaefer^6^,^7^ established different types of typical value ranges (TVR) in UDS measurements, such as initial intravesical pressure (P_ves_), initial abdominal pressure (P_abd_), and initial detrusor pressure (P_det_). Depending on the patient’s testing position, the TVR value of P_ves_ is approximately 10 – 30 cmH_2_O when seated, 5 – 15 cmH_2_O in the lithotomy position, and 25 – 50 cmH_2_O when standing. Using these TVR values, the urodynamicist can determine whether the initial quality of the test is satisfactory.

Statistical process control (SPC) is a methodological approach that employs statistical techniques to monitor, control, and improve processes. Originally developed in the manufacturing industry, SPC has proven to be a versatile tool for ensuring quality consistency and reducing variability. The fundamental principles of SPC involve the use of control charts, which track process performance over time. These charts help identify trends, variations, and deviations from established standards, enabling timely interventions. In the medical field, SPC is increasingly adopted to enhance patient care, optimize clinical workflows, and ensure compliance with regulatory standards. Numerous studies support the application of SPC in healthcare. For example, Benneyan *et al*.^8^ demonstrated the effectiveness of SPC in reducing medication errors in a hospital setting. Similarly, Thor *et al*.^9^ highlighted the role of SPC in improving surgical outcomes through continuous monitoring of procedural adherence and patient recovery times.

In the traditional quality review method, greater emphasis is often placed on the incidence of single or multiple artifacts, rather than assessing whether the entire process is under control. Our center has established the SPC urodynamics quality control approach as a novel strategy for reviewing both the incidence of artifacts and the overall performance of the UDS process.^10^ In a previous study, we introduced SPC for the quality review of UDS over a specific period, transforming the data into visual representations.^11^ However, that study focused solely on the application of SPC to data on artifact occurrence rates (binary categorical variables). In the UDS process, numerous continuous variables require quality control, one of which is the aforementioned TVR value. Therefore, this study aimed to validate the utility of SPC technology for continuous variables such as TVR.

## 2. Methods and materials

### 2.1. Inclusion and exclusion criteria

**Figure 1:**
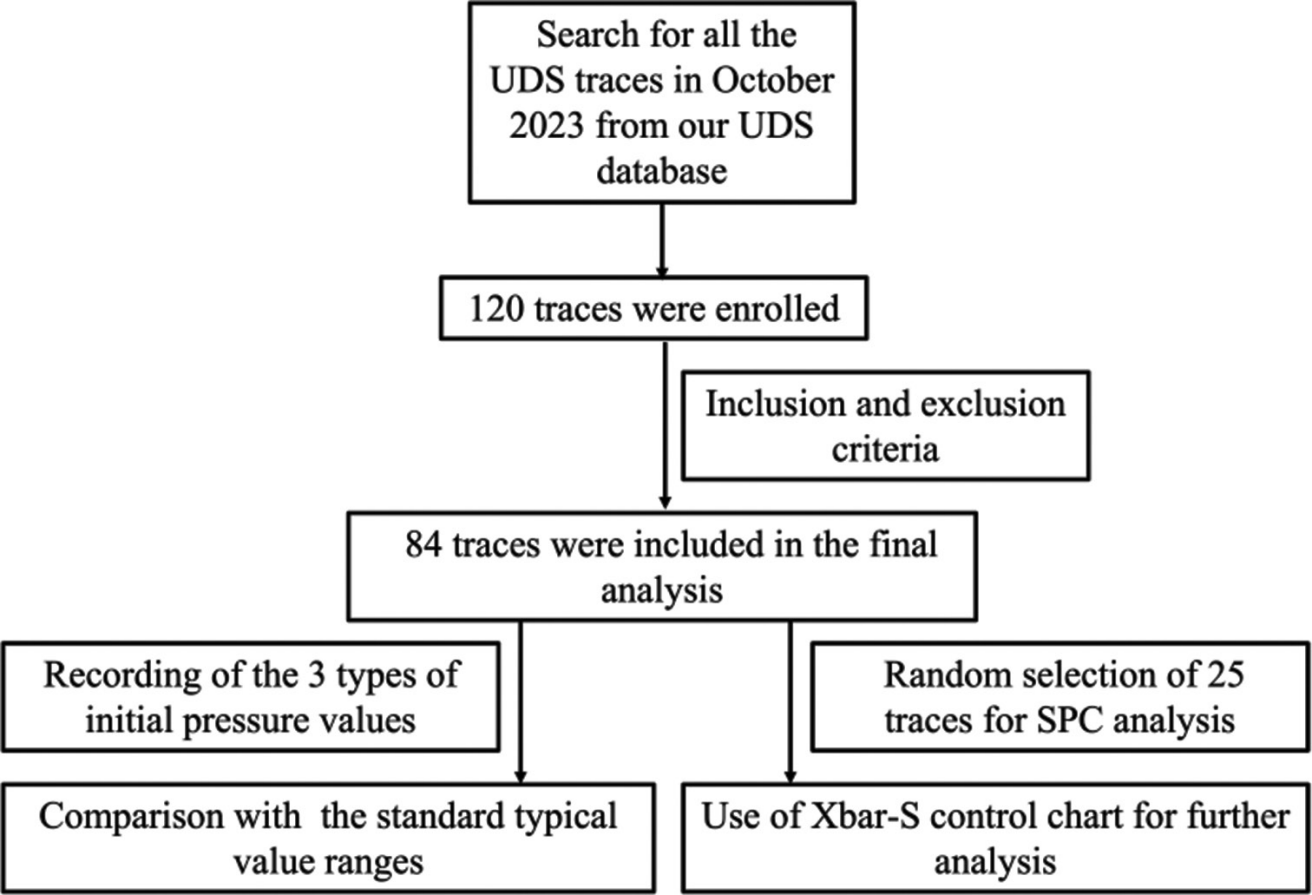
Data selection process. Abbreviations: SPC: Statistical process control; UDS: Urodynamic study; Xbar-S: Mean-standard deviation control chart.

This single-center and retrospective study was conducted in the Department of Urology at West China Hospital of Sichuan University in October 2023. Out of 120 UDS traces collected in October, only 84 met all inclusion and exclusion criteria for final analysis. The initial P_ves_, P_abd_, and P_det_ were recorded for each enrolled UDS trace. The inclusion criteria were as follows: (i) clear and easily recognizable UDS traces, (ii) complete medical history, (iii) signed informed consent from patients, and (iv) use of an air-charged system for UDS. The exclusion criteria included: (i) age <18 years, and (ii) non-standard zero setting was not performed (Figure 1).

### 2.2. UDS process

All UDS were performed using the Laborie Triton Air-charged system (Laborie and Co, Canada) with the matching catheters, following the GUP guidelines. Sterile saline (37°C) was used as the filling medium. For patients with non-neurogenic bladder dysfunction, the filling rate was 61 mL/min, while for patients with neurogenic bladder dysfunction, the filling rate was 11 mL/min. All patients were in a sitting position during the procedure, and the UDS was conducted strictly in accordance with GUP guidelines.^1^

### 2.3. Different categories of UDS artifacts

An urodynamic artifact is defined as any image change that may affect the interpretation of urodynamic results during the examination, due to either technical or non-technical issues.^12^ These artifacts are categorized into discrete and continuous variable data.^13^ Discrete variable data include issues such as non-standard zero settings, the absence of a cough test, and incomplete recording of all UDS measurements.^12^ Continuous variable data refer to the amplitude of transvesical pressure in UDS measurements.

### 2.4. Calculation of initial pressures for enrolled UDS traces and comparison with standard TVR

The average values of initial P_ves_, P_abd_, and P_ves_ were calculated for all 84 enrolled UDS traces. These data were then presented using the scatter diagrams. The TVR were marked on each scatter diagram with reference lines (TVR for initial P_ves_ was 35.4 ± 10.7 cmH_2_O, for initial P_abd_ was 33.1 ± 10.9 cmH_2_O, and for initial P_det_ was 2.3 ± 3.5 cmH_2_O).^6^,^7^

### 2.5. Different categories of Shewhart charts

**Figure 2:**
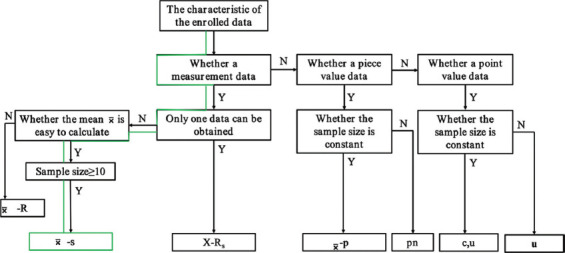
Selection of the mean-standard deviation control charts. Abbreviations: c: Defect numerical control chart; N: No; p: Non-conforming product rate control chart; pn: Non-conforming product numerical control chart; U: Unit defect numerical control chart; x̅-R: Mean-range control chart; x̅-s: Mean-standard deviation control chart; X-R_S_: Single value moving range control chart; Y: Yes.

The Shewhart control chart, first proposed by Dr. W. A. Shewhart of Bell Telephone Laboratories in the United States in 1924, has since become an important tool for scientific management. This chart, which includes control limits (CLs), is used to distinguish whether quality fluctuations are due to random (accidental) or systematic factors.^14^ Shewhart charts include four types of measurement control charts: the mean-range control chart (Xbar-R), the mean-standard deviation control chart (Xbar-S), the median-range control chart (Xmed-R), and the single-value-moving range control chart (X-R_m_). In addition, there are four types of counting control charts: the non-conforming product rate control chart, the non-conforming product numerical control chart, the defect numerical control chart, and the unit defect numerical control chart (Figure 2).^10^,^15^,^16^

### 2.6. Fundamental theory of the mean-standard deviation control chart

The Xbar-S chart is a commonly used data control chart, consisting of a mean (X-bar) chart and a standard deviation (S) chart. The X-bar chart primarily assesses the stability of the production process mean, while the S chart evaluates the stability of the standard deviation. Conventionally, the X-bar chart is placed above the S chart. The Xbar-S chart is typically employed when the sample size per subgroup exceeds five.^17^

### 2.7. Calculation formulas for the mean-standard deviation control chart and defining the abnormal fluctuations in SPC charts

We randomly selected 25 cases from the 84 enrolled cases for SPC (Xbar-S control chart) analysis. The following formulas were used to calculate the CL, upper CL (UCL), and lower CL (LCL) for the X-bar chart and S chart:

The formulas for X-bar chart:


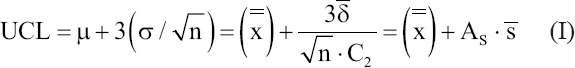



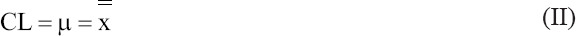



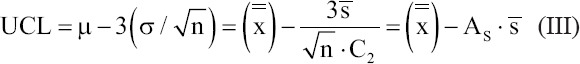



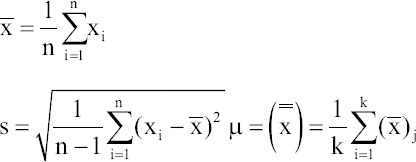


*According to the constant table, A3=0.606 when the sample size is 25.

The formulas for S chart:


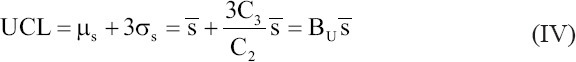



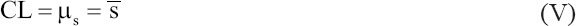



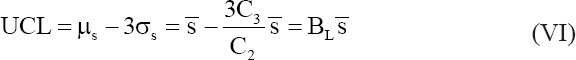



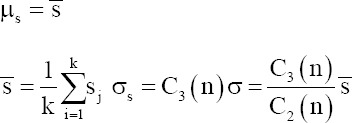


*According to the constant table, B_U_=1.435, B_L_=0.565 when the sample size is 25.

## 3. Results

### 3.1. Characteristics of enrolled data

**Figure 3:**
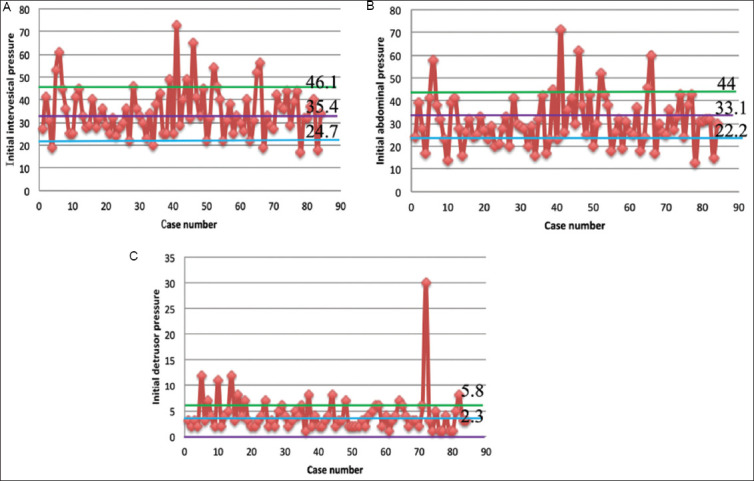
Scatter diagram for each initial pressure compared with standard typical value range (TVR) Notes: The purple line represents the mean of the standard TVR, the green line represents mean+standard deviation, and the blue line denotes mean-standard deviation. (A) Initial P_ves_ versus the standard TVR for P_ves_; (B) Initial P_abd_ versus the standard TVR for P_abd_; (C) Comparison of initial P_det_ with the standard TVR for P_det_. Abbreviations: P_abd_: Initial abdominal pressure; P_det_: Initial detrusor pressure; P_ves_: Initial intravesical pressure.

A total of 84 urodynamic traces that satisfied the inclusion and exclusion criteria were included in the analysis. Of these, 20 were female, and 64 were male, with an average age of 58.02 ± 16.09 years. Thirty-two cases were diagnosed with neurogenic bladder dysfunction, and 52 with non-neurogenic bladder dysfunction. The average initial P_ves_ was 34.81 ± 10.78 cmH_2_O, the initial P_abd_ was 30.92 ± 11.14 cmH_2_O, and the initial P_det_ was 4.20 ± 3.73 cmH_2_O. Scatter diagrams were subsequently employed to visualize these data for further analysis. Each diagram included a standard TVR mark line (standard TVR for initial P_ves_: 35.4 ± 0.7 cmH_2_O, standard TVR for initial P_abd_: 33.1 ± 0.9 cmH_2_O, and standard TVR for initial P_det_: 2.3 ± 3.5 cmH_2_O) (Table 1 and Figure 3).

**Table 1:** Characteristics of the included data

Characteristic	Value
Total participants	84
Gender	
Male, *n* (%)	64 (76)
Female, *n* (%)	20 (24)
Age (years), mean±standard deviation	58.02±16.09
Neurogenic bladder dysfunction, *n* (%)	
Yes	32 (38)
No	52 (62)
Average initial pressure (cmH_2_O), mean±standard deviation	
Intravesical pressure	34.81±10.78
Abdominal pressure	30.92±11.14
Detrusor pressure	4.20±3.73

### 3.2. Mean-standard deviation control chart calculation results for enrolled 25 cases

**Figure 4:**
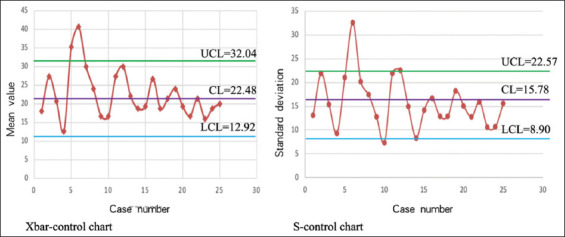
X-bar control chart and S-control chart for 25 enrolled cases Abbreviations: CL: Control limit; LCL: Lower control limit; UCL: Upper control limit.

The CL for the X-bar control chart was 22.48, with the UCL at 32.04 and the LCL at 12.92. For the S-control chart, the CL was 15.78, the UCL was 22.57, and the LCL was 8.9. These data were then used to plot separate X-bar control and S control charts. Based on the criteria for defining abnormal fluctuations, three such fluctuations were observed in these control charts, suggesting potential process instability.^8^,^18^-^20^ Specifically, two cases exceeded the UCL in the X-bar control chart, while one case was above the UCL in the S control chart, all other cases remaining within the CLs (Figure 4).

## 4. Discussion

Traditional quality assessment of UDS often requires substantial human and financial resources to identify quality issues. SPC offers a straightforward method to assess UDS quality using CLs. It helps determine process stability and detects abnormalities. Several factors contribute to abnormal fluctuations in UDS TVRs, including: (i) Executives: The technical proficiency and educational background of urodynamicists; (ii) UDS machine: Ensuring the equipment meets industry standards and is regularly calibrated; (iii) Method: The use of appropriate quality control methods; and (iv) Environment: Factors such as ambient lighting, layout, and temperature. From a quality control perspective, these factors can be categorized into common causes (accidental) and special causes (systematic). Common causes are universal factors affecting quality, while special causes occur under abnormal circumstances, significantly impacting process quality. Distinguishing between these causes based on experience alone is challenging, underscoring the need for SPC methods in practical quality control processes.

Liao and Schaefer^6^,^7^ have defined various types of TVRs for quality control in UDS. These TVRs are integral to assessing overall quality during UDS procedures. For instance, after the initial zero-setting process in UDS, the TVR for initial P_ves_, initial P_abd_, and initial P_det_ are used to evaluate whether pressures fall within an acceptable and high-quality range. In our study, we compared our measured initial pressures with the standard TVRs: initial P_ves_ (34.81 ± 10.78 cmH_2_O versus 35.4 ± 0.7 cmH_2_O), initial P_abd_ (30.9 ± 11.14 cmH_2_O versus 33.1 ± 0.9 cmH_2_O), and initial P_det_ (4.20 ± 3.73 cmH_2_O versus 2.3 ± 3.5 cmH_2_O). We further employed visual data processing to convert these comparisons into graphical form, delineating the quality control upper and lower limits on the image based on the TVR. It is important to note that this chart differs from the SPC control charts discussed later, and the upper and lower limits mentioned here are different from the UCL and LCL in SPC charts. While no significant differences were observed between our measured pressures and their respective standard TVRs, the scatter diagram indicated fluctuations that exceeded the upper limits, suggesting the occurrence of abnormal causes as described earlier. Further analysis is warranted to investigate these issues.

To further analyze the quality of UDS at the start of the procedure, we used Xbar-S control charts to visualize the data. Separate X-bar control and S-control charts were plotted, revealing that two cases exceeded the UCL in the X-bar control chart, while one case went beyond the UCL in the S control chart. However, the data points were generally more uniformly distributed within the CLs, suggesting that while there were some issues affecting process quality at the outset of UDS, overall performance remained acceptable. When we considered the scatter diagram generated earlier, we observed outliers beyond the control lines. These outliers likely contributed to the abnormal fluctuations observed in the SPC chart.

This study is subject to several limitations. The study’s sample size was relatively small, and the selection criteria may not fully represent all potential variations in UDS procedures. In addition, the findings may not be universally applicable due to variations in patient demographics, clinical practices, and equipment used across different healthcare settings. The accuracy of measurements, particularly in capturing initial pressures and interpreting scatter diagram data, may have been influenced by human error or technological limitations. Moreover, conducting the study at a single center limits the diversity of clinical practices and patient populations considered, which could impact the study’s external validity. Addressing these limitations in future research could enhance the robustness and generalizability of findings regarding the application of SPC in UDS.

## 5. Conclusion

The clinical utility of SPC in assessing the quality of UDS using binary categorical variables has been established in prior research. This study provides initial verification of the application of SPC to continuous variable data, such as TVR in UDS parameters. Furthermore, we highlight the pivotal role of TVR in UDS quality control. The results of this study should be further validated in a larger, multi-center, and prospective study.

## Data Availability

The data supporting the findings of this article are available from the corresponding author on reasonable request.

## References

[1] Rosier PF Schaefer W Lose G International Continence Society Good urodynamic practices and terms 2016:Urodynamics, uroflowmetry, cystometry, and pressure-flow study. Neurourol Urodyn 2017;36 (5):1243–1260. doi: 10.1002/nau.23124 27917521 10.1002/nau.23124

[2] Reitz A Knapp PA Frey S Schurch B Functional magnetic stimulation of the spinal cord-a urodynamic study in healthy humans. Neurourol Urodyn 2004;23:148–153. doi: 10.1002/nau.20014 14983427 10.1002/nau.20014

[3] Blaivas JG The bladder is an unreliable witness. Neurourol Urodyn 1996;15 (5):443–445. doi: 10.1002/(SICI)1520-6777(1996)15:5<443:AID-NAU1>3.0.aCO;2-F 8857612 10.1002/(SICI)1520-6777(1996)15:5<443::AID-NAU1>3.0.CO;2-F

[4] Zeng X Xia Z Peng L Quality of urodynamics:A national cross-sectional study in china. Chin Med J 2023;136:236–238. doi: 10.1097/CM9.0000000000002203 36952621 10.1097/CM9.0000000000002203 PMC10106133

[5] Gammie A Clarkson B Constantinou C International Continence Society guidelines on urodynamic equipment performance. Neurourol Urodyn 2014;33:370–379. doi: 10.1002/nau.22546 24390971 10.1002/nau.22546

[6] Liao LM Schaefer W Urodynamic quality control (I):Establishment of TVR and its role in real-time quantitative quality control. Chin J Urol 2006;27 (5):4

[7] Liao LM Schaefer W Urodynamic Quality Control (II):Recognition of typical signal patterns and its role in real-time qualitative quality control. Chin J Urol 2006;27 (5):4

[8] Benneyan JC Lloyd RC Plsek PE Statistical process control as a tool for research and healthcare improvement. Qual Saf Health Care 2003;12 (6):458–464. doi: 10.1136/qhc.12.6.458 14645763 10.1136/qhc.12.6.458 PMC1758030

[9] Thor J Lundberg J Ask J Application of statistical process control in healthcare improvement:Systematic review. Qual Saf Health Care 2007;16 (5):387–399. doi: 10.1136/qshc.2006.022194 17913782 10.1136/qshc.2006.022194 PMC2464970

[10] Zeng X Shen SH Shen H Statistical process control for the analysis of quality control in urodynamics:A potential new approach for quality review of urodynamics. Neurourol Urodyn 2023;42:289–296. doi: 10.1002/nau.25081 36321794 10.1002/nau.25081

[11] Vetter TR Morrice D Statistical process control:No hits, no runs, no errors?. Anesth Analg 2019;128 (2):374–382. doi: 10.1213/ane.0000000000003977 30531221 10.1213/ANE.0000000000003977

[12] Zeng X Wu J Luo D Urodynamics quality in southwest China:A multicenter random study. Chin J Urol 2021;12:455–461

[13] Nguyen HO Tongzon J Application of the discrete variable investment model to analyse the decision to adopt e-business among transport and logistics companies. Int J Log Res Appl 2012;15:251–267. doi: 10.1080/13675567.2012.741221

[14] Jing Z Liang G The Analysis of shewhart control chart in remanufacture. Machine Tool &Hydraulics 2009;21:123–127

[15] Linna KW Improving Shewhart control chart performance in the presence of measurement error using multiple measurements and two-stage sampling. J Int Interdiscip Bus Res 2018;5:10. doi: 10.58809/LVIF9073

[16] Yasui S Ojima Y Suzuki T Generalization of the Run Rules for the Shewhart Control Charts. Germany:Physica-Verlag HD 2006. doi: 10.1007/3-7908-1687-6_13

[17] Stuart M Mullins E Drew E Statistical quality control and improvement. Eur J Oper Res 1996;88 (2):203–214. doi: 10.1016/0377-2217(95)00069-0

[18] Pereira P Seghatchian J Caldeira B Xavier S de Sousa G Statistical methods to the control of the production of blood components:Principles and control charts for variables. Transfus Apheresis Sci 2018;57:132–142. doi: 10.1016/j.transci.2018.02.022. 10.1016/j.transci.2018.02.022 29526479

[19] Lv F Wang H Kong D. Quality Management in Pellet Feed Mill Based on Statistical Process Control (SPC). United States:ASABE;2016. doi: 10.13031/aim.20162459436

[20] Zhang TJ Chen CM Yi Z Statistical quality control of cosmetics. China Surfactant Deterg Cosmet;2013:37–40

